# Infective endocarditis meets native vertebral osteomyelitis: a mortality perspective

**DOI:** 10.5194/jbji-10-425-2025

**Published:** 2025-11-05

**Authors:** Fabio Borgonovo, Francesco Petri, Takahiro Matsuo, Rita Igwilo-Alaneme, Seyed Mohammad Amin Alavi, Omar K. Mahmoud, Said El Zein, Matteo Passerini, Mohammad Hassan Murad, Daniel C. DeSimone, Ahmad Nassr, Aaron J. Tande, Andrea Gori, Elie F. Berbari

**Affiliations:** 1 Department of Infectious Diseases, ASST Fatebenefratelli Sacco, “L. Sacco” University Hospital, 20157 Milan, Italy; 2 Division of Public Health, Infectious Diseases and Occupational Medicine, Department of Medicine, Mayo Clinic College of Medicine and Science, Mayo Clinic, Rochester, MN 55905, USA; 3 Faculty of Medicine, Ahvaz Jundishapur University of Medical Sciences, 6135715753 Ahvaz, Iran; 4 Department of Pathophysiology and Transplantation, University of Milan, Milan, Italy; 5 Evidence-Based Practice Center, Mayo Clinic, Rochester, Minnesota, USA; 6 Department of Orthopedic Surgery, Mayo Clinic, Rochester, Minnesota, USA; 7 Centre for Multidisciplinary Research in Health Science (MACH), University of Milan, 20122 Milan, Italy

## Abstract

**Background**: Native vertebral osteomyelitis and infective endocarditis (NVO + IE) are increasingly recognized as overlapping entities, sharing common risk factors (e.g., advanced age, immunosuppression) and similar pathogen profiles, most commonly *Staphylococcus aureus* and streptococci. Concurrent infection presents unique diagnostic and therapeutic challenges, leading to uncertainty regarding clinical outcomes and mortality. Therefore, we aimed to systematically evaluate the combined mortality associated with concomitant NVO + IE and to summarize the available clinical characteristics from published studies. **Methods**: A systematic review was conducted following the PRISMA framework. The databases searched included MEDLINE, Embase, Cochrane Library, and Scopus from 1970 to October 2023. Studies were included if they involved at least 10 adult patients diagnosed with NVO and IE and provided mortality data. Two reviewers independently screened the references, extracted the data, and evaluated the methodological quality using a dedicated tool. A random-effects meta-analysis was performed to aggregate in-hospital, 1-month, 1-year, and 3-year mortality rates. **Results**: A total of 16 studies (12 retrospective, 3 prospective, 1 mixed) were included, involving 641 patients (mean age 67.1 years) with NVO + IE. In-hospital mortality was 14.0 % (95 % CI: 10.0 %–20.0 %). At 1 month, mortality was 9.0 % (95 % CI: 5.0 %–17.0 %), rising to 18.0 % (95 % CI: 13.0 %–24.0 %) by 1 year and 16.0 % (95 % CI: 3.0 %–50.0 %) by 3 years. Significant between-study heterogeneity was observed ( $I^{2}$ range: 3 %–70 %). Common co-morbidities included diabetes mellitus (23.7 %), chronic renal failure (15.0 %), and immunosuppression (15.0 %). Streptococci (31.5 %), *S. aureus* (25.2 %), and enterococci (17.7 %) were the primary pathogens. Cardiac valve surgery and spinal surgery were reported in 47.5 % and 29.9 % of patients, respectively. A subgroup analysis on 1-month mortality showed that *S. aureus* predominance was associated with a significantly higher mortality compared to streptococci. Certainty in the estimates was low due to imprecision and methodological limitations. **Conclusions**: Concomitant NVO + IE is associated with substantial mortality, especially for *S. aureus*, underscoring the need for earlier diagnosis, coordinated multidisciplinary management, and standardized treatment protocols. Future prospective, high-quality studies are needed to clarify optimal strategies for diagnostic workup and surgical intervention for this complex clinical scenario.

## 1 Introduction

Native vertebral osteomyelitis (NVO) is a serious infection of the spine with an incidence that has been steadily rising in the United States (Talha et al., 2022; Issa et al., 2018; Kremers et al., 2015). Despite advancements in diagnostic and therapeutic strategies, clinical outcomes remain poor (McHenry et al., 2002), highlighting the need for more effective management approaches. Current practices are predominantly guided by the 2015 Infectious Diseases Society of America (IDSA) guidelines (Berbari et al., 2015), though recent research continues to expand our understanding of this condition.

Infective endocarditis (IE) incidence has increased substantially over the past 3 decades, yet mortality rates have remained consistently high, underscoring the persistent burden of this condition (Mettler et al., 2023). In response to recent evidence, updated definitions and management guidelines have been proposed, reflecting progress in the field (Fowler et al., 2023).

There is a biologically plausible overlap between NVO and IE (NVO + IE), as both conditions share similar risk factors, such as advanced age, immunosuppression, and diabetes mellitus, as well as a comparable range of pathogens, notably *Staphylococcus aureus* (Douiyeb et al., 2025). These shared characteristics create diagnostic and therapeutic complexities, particularly in distinguishing the primary site of infection and the timing of acquisition. As a result, the number of patients presenting with concomitant NVO + IE is expected to rise, presenting an increasingly common clinical scenario (Douiyeb et al., 2025).

Despite this growing recognition, current literature is fragmented and dominated by low-quality evidence, such as small retrospective cohort studies. This limitation has hindered our ability to understand pathogen patterns, optimize early recognition and management of these concurrent infections, and accurately describe patient outcomes. Notably, it remains unclear whether the co-occurrence of NVO + IE results in higher mortality or poorer prognoses compared to either condition alone.

To address this gap, we aim to conduct a systematic review of the literature to summarize the combined mortality rate associated with these two conditions.

## 2 Material and methods

### 2.1 Methods

This systematic review was reported in accordance with the Preferred Reporting Items for Systematic Reviews and Meta-Analyses (PRISMA) statement (Page et al., 2021).

### 2.2 Protocol

An a priori protocol was developed prior to initiating this study. The protocol is available upon request from the first and/or corresponding author.

### 2.3 Eligibility criteria

We included studies that reported on at least 10 adults with NVO + IE co-infection and reported mortality in terms of absolute numbers or proportions. The threshold of 10 patients was chosen to balance data generalizability with the feasibility of conducting a focused and methodologically robust systematic review. Detailed inclusion and exclusion criteria are outlined in the Supplement. Case reports were excluded to reduce publication bias. Study protocols, trial registrations, secondary analyses, commentaries, reviews, and conference proceedings were also excluded.

The primary outcome was mortality in patients with NVO + IE co-infection. A secondary objective was to identify the limited clinical characteristics of such patients available from published studies.

### 2.4 Data sources and search strategies

A comprehensive search of several databases was performed on 19 October 2023. No date or language restrictions were applied to the search. Databases (and the dates of content coverage) were Ovid MEDLINE(R) ( 1946+ including epub ahead of print, in-process, and other non-indexed citations), Ovid Embase ( 1974+ ), Ovid Cochrane Central Register of Controlled Trials ( 1991+ ), Ovid Cochrane Database of Systematic Reviews ( 2005+ ), and Scopus via Elsevier ( 1970+ ).

The search strategies were designed and conducted by a medical librarian with input from the study investigators. Controlled vocabulary supplemented with keywords was used. The actual strategies listing all search terms used and how they are combined are available in the Supplement.

### 2.5 Study selection

Abstract and full-text screening were managed using the Covidence Systematic Review Software (Veritas Health Innovation, Melbourne, Australia). Three independent reviewers (FB, FP, OM) conducted the screening process, with each article reviewed by two of them. Disagreements were resolved through consultation with a third reviewer and consensus discussions to maintain consistency and minimize bias in the selection process.

Studies that included patients with NVO + IE as a subgroup were also evaluated, but the mortality rate of NVO + IE was usually not reported. We therefore contacted the corresponding authors of 56 studies. Of the 56 emails sent, 13 were returned as undeliverable. Despite follow-up attempts, 31 authors did not respond. Among the responses received, 6 authors indicated that the requested data were no longer available, and 2 clarified that their cohorts included fewer than 10 patients with NVO + IE, rendering their data ineligible for inclusion. Ultimately, 4 authors provided the requested data, all of which included cohorts with more than 10 patients with NVO + IE and were considerable suitable for analysis.

### 2.6 Data extraction

A data extraction form was developed to ensure clarity and appropriateness for capturing relevant information. The form was initially tested by two reviewers (FB and FP) and subsequently refined based on input from the research team. This iterative process ensured clarity and minimized potential misunderstandings during data collection. Following this, two reviewers (FB and FP) extracted relevant information from each article, recording the data in separate Excel sheets.

Extracted variables included study characteristics (author names, year of publication, journal name, funding source, study location, study period, study design, research question, and sample size), clinical characteristics of patients with NVO + IE (age, sex, co-morbidities, microbiology, and treatment details), and mortality outcomes. Predictors of mortality extracted included methicillin-sensitive *S. aureus* (MSSA) and methicillin-resistant *S. aureus* (MRSA) status, as well as treatment modality (surgical vs. conservative).

Methodological quality was conducted using the modified Newcastle–Ottawa scale (NOS) for case reports and case series by two authors (FB and FP) (Murad et al., 2018). A comprehensive list of extracted data is available in the Supplement. Certainty in the pooled estimates was assessed using the GRADE approach adaptation for prognosis (Spencer et al., 2012).

### 2.7 Statistical analysis

The main outcome evaluated was the mortality rate, expressed as a single proportion. When available, data on overall in-hospital mortality and all-cause mortality at 28–30 d (or 1 month), 1 year, and 3 years were extracted from the studies. For continuous variables, the analysis presented the median and interquartile range, while frequencies were reported for categorical variables, averaging or calculating values across studies without meta-analysis. The sample mean was estimated from the median and interquartile range according to previous works (Wan et al., 2014). To obtain a pooled mortality rate, a random-effects meta-analysis was performed because of the heterogeneity in patient characteristics and study setting using logit transformation to stabilize variance, with forest plots illustrating both individual study results and the overall summary estimate with 95 % confidence intervals. The $I^{2}$ statistic was chosen to assess heterogeneity. Publication bias was assessed through visual inspection of funnel plots. A random-effects subgroup meta-analysis was conducted to compare 1-month mortality according to the predominant pathogen across all studies (*Staphylococcus aureus* vs. *Streptococcus* spp.; see Table 1). Given the presence of studies with zero events, the Freeman–Tukey double arcsine transformation was preferred over the logit transformation to improve model stability for this subgroup analysis. Studies with insufficient data to assign a predominant pathogen were excluded from this analysis. This outcome was selected as it was the most commonly reported across studies. WebPlotDigitizer (https://automeris.io, last access: 1 July 2025) was used to help reviewers to recover raw data from chart images. The statistical analysis utilized BlueSky Statistics software v.10.3 (BlueSky Statistics LLC, Chicago, IL, USA).

**Table 1:** Clinical characteristics of the patients with NVO + IE.

Characteristic	No. of	Total no.	N (%)
	studies	of patients	
		NVO + IE	
Age (mean, years)	14	460	67.1
Females	15	496	122 (24.6)
Diabetes mellitus	8	312	74 (23.7)
Immunosuppressed	5	233	35 (15.0)
Chronic renal failure	8	334	50 (15.0)
Predisposing heart condition	7	176	70 (39.8)
Prosthetic valve	6	248	62 (25.0)
Presence of emboli	9	323	119 (36.8)
Presence of epidural abscess	7	283	48 (17.0)
Microbiology – *S. aureus*	15	496	125 (25.2)
Microbiology – streptococci	14	460	145 (31.5)
Microbiology – enterococci	13	446	79 (17.7)
Vertebral level – cervical	8	297	45 (15.2)
Vertebral level – thoracic	8	297	61 (20.5)
Vertebral level – lumbar/sacral	8	297	180 (60.6)
Management – surgery for IE	10	362	172 (47.5)
Management – surgery for NVO	5	264	79 (29.9)

**Figure 1:**
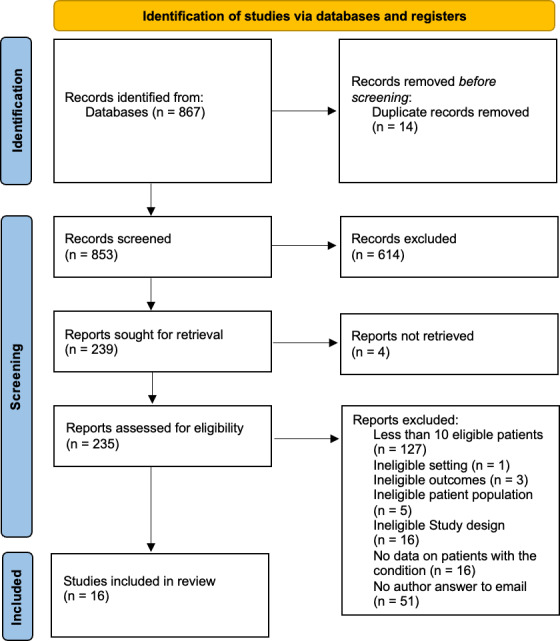
PRISMA 2020 flowchart.

## 3 Results

### 3.1 Study and population characteristics

The literature search yielded 867 studies (Fig. 1). After abstract and full-text screening, 16 studies were included, comprising 12 retrospective investigations, 3 prospective investigations, and 1 mixed investigation (Table S4). Most were conducted in Europe (13/16, 81.2 %), and a total of 641 patients with concomitant NVO + IE were analyzed. Females accounted for 24.6 % (122/496) of cases, and the pooled mean age was 67.1 years. Co-morbidities were common: diabetes mellitus in 23.7 % (74/312), chronic renal failure in 15.0 % (50/334), and immunosuppression in 15.0 % (35/233). Predisposing heart conditions occurred in 39.8 % (70/176), and prosthetic cardiac valves occurred in 25.0 % (62/248). Infectious complications included embolic events in 36.8 % (119/323) and epidural abscesses in 17.0 % (48/283). Streptococci were the most frequent pathogens (31.5 %, 145/460), followed by *Staphylococcus aureus* (25.2 %, 125/496) and enterococci (17.7 %, 79/446). Vertebral involvement was predominant in the lumbar/sacral region (60.6 %, 180/297), with thoracic (20.5 %, 61/297) and cervical (15.2 %, 45/297) sites less often affected. Surgical intervention was performed in 47.5 % (172/362) for IE and in 29.9 % (79/264) for NVO (Table 1).

### 3.2 In-hospital mortality

**Figure 2:**
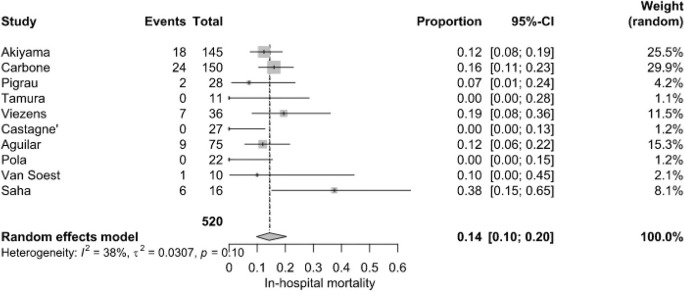
In-hospital mortality of patients with NVO + IE.

Early mortality among hospitalized patients was 14.0 % (95 % CI: 10.0 %–20.0 %), based on 67 deaths among 520 patients from 10 studies. However, individual studies reported wide variations, ranging from 0 % to 37.5 %. Saha et al. (2022) recorded the highest in-hospital mortality (37.5 %), while Castagné et al. (2021), Tamura et al. (2010), and Pola et al. (2018) reported the lowest (0 %). Moderate heterogeneity ( $I^{2}=38$ %, P=0.1 ) was recorded (Fig. 2).

#### 3.2.1 Month mortality

**Figure 3:**
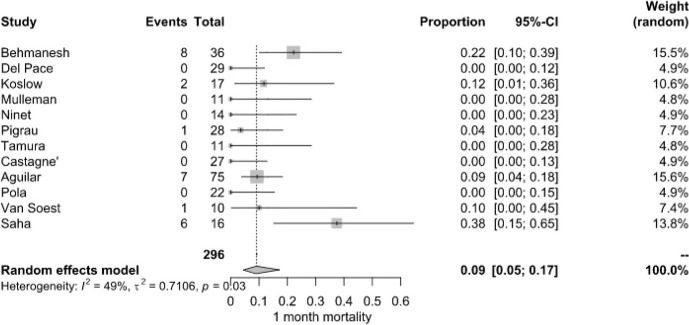
1-month mortality of patients with NVO + IE.

Short-term survival outcomes demonstrated a pooled mortality rate of 9.0 % (95 % CI: 5.0 %–17.0 %), derived from 25 deaths among 296 patients across 10 studies. Mortality rates at this stage were more variable, with some studies recording no deaths, while Behmanesh et al. (2019) observed the highest rate (22.2 %). Heterogeneity remained moderate ( $I^{2}=49$ %, P=0.03 ) (Fig. 3).

#### 3.2.2 Year mortality

**Figure 4:**
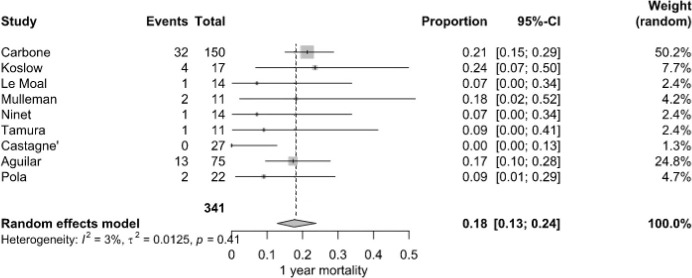
1-year mortality of patients with NVO + IE.

By the end of the first year, the mortality rate increased to 18.0 % (95 % CI: 13.0 %–24.0 %), with 56 deaths among 341 patients from nine studies. Carbone et al. (2020) (21.3 %) and Koslow et al. (2014) (23.5 %) reported markedly higher rates than Castagné et al. (2021) (0 %). Heterogeneity was considered low ( $I^{2}=3$ %, P=0.41 ) (Fig. 4).

#### 3.2.3 Year mortality

**Figure 5:**
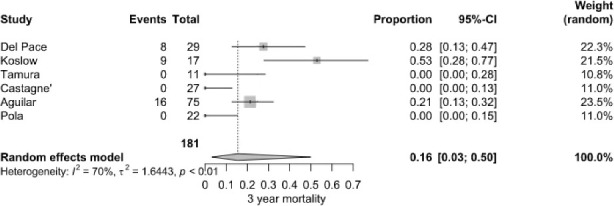
3-year mortality of patients with NVO + IE.

The pooled 3-year mortality rate was 16.0 % (95 % CI: 3.0 %–50.0 %), based on 33 deaths among 181 patients from six studies. Koslow et al. (2014) reported an exceptionally high rate (52.9 %), whereas several other studies documented rates below 10 %. High heterogeneity was calculated ( $I^{2}=70$ %, P<0.001 ) (Fig. 5).

### 3.3 Publication bias

Funnel plot inspection did not show substantial asymmetry across analyses, suggesting no clear evidence of publication bias (Table S1 in the Supplement). The distribution of studies appeared reasonably symmetrical around the pooled estimates, particularly in analyses with ≥10 studies. However, due to the limited number of included studies in some models, the ability to formally detect small-study effects or reporting bias was limited.

### 3.4 Subgroup analysis on predominant pathogen

**Figure 6:**
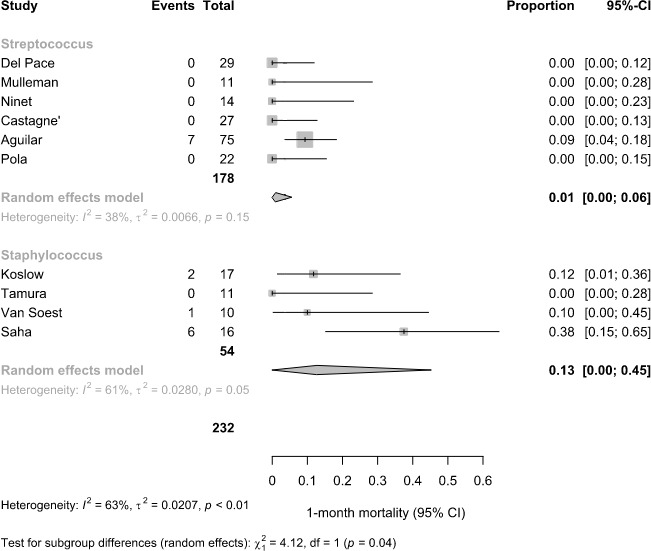
Subgroup analysis of 1-month mortality, based on the predominant pathogen reported across the studies.

In the subgroup analysis of 1-month mortality, studies were classified based on the predominant pathogen reported (Fig. 6). Among six studies with a predominance of *Streptococcus* spp., the pooled 1-month mortality was 1 % (95 % CI: 0 %–6 %; $I^{2}=38$ %). In contrast, in four studies reporting *Staphylococcus* spp. as the predominant pathogen, the pooled mortality was higher at 13 % (95 % CI: 0 %–45 %; $I^{2}=61$ %). The difference between subgroups was statistically significant ( $\chi^{2}=4.12$ , df = 1, p=0.04 ), suggesting a potential pathogen-related effect on short-term mortality.

### 3.5 Methodological quality and certainty of evidence

The majority (10/16, 62.5 %) of the studies were deemed to have acceptable methodological quality (Table S2). The overall certainty of evidence was deemed low due to the serious concerns about methodological limitations and imprecision (Table S8).

## 4 Discussion

In this systematic review, we analyzed the combined mortality in patients with concomitant NVO + IE across multiple studies. Mortality remained substantial, with an in-hospital rate of 14.0 %, 9.0 % at 1 month, 18.0 % at 1 year, and 16.0 % at 3 years. For context, the annual mortality rate in a healthy US cohort aged around 67 years is approximately 2 %–3 % (United States Social Security Administration, 2025), underscoring the significantly elevated risk in patients with NVO + IE (Weber et al., 2024). These findings highlight the long-term burden of this dual pathology and reinforce the need for earlier diagnosis and optimized management strategies. One study (Saha et al., 2022) reported exceptionally high in-hospital mortality (37.5 %). We did not perform sensitivity analysis due to the low number of patients and because we think that this outlier would not materially change the pooled estimates. Nonetheless, the unexplained heterogeneity across studies suggests variability in patient populations, clinical approaches, and follow-up durations.

The dual burden of NVO + IE represents a complex clinical entity requiring careful attention. Both infections share risk factors such as advanced age, diabetes, and immunosuppression, yet their simultaneous occurrence poses unique challenges (Douiyeb et al., 2025; Koslow et al., 2014). Diagnostic uncertainty remains a critical issue, as distinguishing primary infection sites and determining the optimal treatment strategy often lead to delays in intervention. Furthermore, the high prevalence of embolic complications (26.6 %) and epidural abscesses (22.8 %) may contribute to worse outcomes, emphasizing the need for early recognition and aggressive management.

The microbiological profile of NVO + IE patients further supports the complexity of this condition. The microbiological profile of combined NVO + IE reflects a distinct pathogen distribution compared with isolated infections. In native-valve endocarditis alone, *Staphylococcus aureus* is the leading pathogen followed by enterococci and streptococci (Talha et al., 2020). In isolated vertebral osteomyelitis, *S. aureus* again predominates ( ≈47 %), streptococcal species account for ≈21 %, and Enterobacteriaceae account for ≈20 %, consistent with IDSA guidelines (Berbari et al., 2015; Kim et al., 2019; Desoutter et al., 2015). By contrast, our pooled NVO + IE data revealed streptococci in 31.5 % of cases, *S. aureus* in 25.2 % of cases, and enterococci in 17.7 % of cases (Douiyeb et al., 2025). These shifts underscore unique treatment challenges, particularly the high risk of antibiotic resistance and biofilm formation on both cardiac valves and vertebral structures. However, it must be noted that our systematic review comprises articles published across a time span of decades, making definitive conclusions on contemporary epidemiology not possible.

The subgroup analysis suggested higher 1-month mortality in studies with *S. aureus* as the predominant pathogen compared to *Streptococcus* spp. This finding aligns with the well-established clinical profile of *S. aureus* as a more aggressive pathogen, often associated with complications such as metastatic infection, embolic phenomena, and a greater need for surgical management (Writing Committee Members et al., 2021; Luehr et al., 2023). In contrast, *Streptococcus* spp. is typically linked to a more indolent disease course and more favorable outcomes when treated appropriately. These results support the clinical relevance of microbiological etiology as a determinant of prognosis in patients with NVO + IE.

Our findings suggest that cardiac valve surgery and spinal surgery were reported in 47.5 % and 29.9 % of patients, respectively, yet its impact on mortality remains uncertain since it was not possible to perform a subgroup analysis due to small sample size. While surgery is often necessary for uncontrolled infection, spinal instability, or neurological deterioration, its timing and indications in the setting of NVO + IE require further investigation. Given the lack of randomized trials on this topic, multidisciplinary decision-making involving infectious disease specialists, cardiologists, and spine surgeons is essential to optimizing patient outcomes.

When comparing our results with prior literature, the mortality observed in NVO + IE appears comparable to or higher than that reported in isolated IE or NVO. Previous studies on IE-related mortality have estimated 1-year rates ranging from 30 % to 40 %, depending on co-morbidities and pathogen virulence, whereas NVO-associated mortality is typically lower (Taduru, 2023; Hammond-Haley et al., 2023). Interestingly, our pooled 1-year mortality estimate for NVO + IE (18 %) was lower than the 30 %–40 % often reported in large, isolated IE cohorts. Several factors may explain this discrepancy. Patients with fulminant IE may have been underrepresented in studies of concomitant NVO + IE, leading to selection bias. Some cohorts excluded early fatal cases or had incomplete follow-up, potentially lowering observed rates. Moreover, the presence of vertebral involvement may have prompted earlier imaging, multidisciplinary consultation, or surgical intervention, indirectly improving survival. These hypotheses remain speculative and highlight the need for prospective comparative studies. Our findings suggest that the combined presence of both infections may confer an additive risk, possibly due to increased inflammatory burden, delayed diagnosis, or more severe clinical presentation. Recent studies provide additional insights into the prognosis and management of patients with NVO + IE. Douiyeb et al. (2025) reported a prevalence of vertebral osteomyelitis in 11.5 % of patients with IE. Their findings indicate that enterococcal infections and advanced age are independent risk factors for concomitant vertebral osteomyelitis, supporting our observation of enterococci as a significant pathogen in our cohort, in accordance with the recent inclusion of enterococci as “typical endocarditis bacteria” regardless of the primary source incorporated in the 2023 Duke-International Society of Cardiovascular Infectious Diseases (ISCVID) criteria for IE diagnosis (Fowler et al., 2023). Importantly, their study found no significant difference in 5-month mortality between patients with and without NVO (21.9 % vs. 19.6 %), suggesting that early detection and optimized management may mitigate excess mortality in these patients. Weber et al. (2024) investigated the impact of surgical sequence in patients with NVO + IE. They found that primary surgery for NVO was associated with significantly higher 30 d mortality (25.7 %) compared to primary surgery for IE (11.4 %), while primary surgical treatment for IE was linked to a higher recurrence rate (12.2 %). This highlights the critical need for a tailored approach to surgical decision-making. In contrast, our meta-analysis was not able to stratify outcomes based on surgical timing and type of surgery, including the use of spinal instrumentation, suggesting that future studies should explore the impact of surgical sequence on long-term mortality and recurrence. Hijazi et al. (2023) provided additional insights into the microbiological and clinical profiles of patients with concomitant IE and spinal infections. Their study reported a higher prevalence of hepatic cirrhosis, septic embolism, and enterococcal infections among patients with IE and primary spinal infections compared to those without IE. Notably, they found that the modified Duke criteria had a sensitivity of 100 % but a specificity of only 66.7 % in diagnosing IE in patients with spinal infections. These findings highlight the potential limitations of current diagnostic criteria in this patient population and reinforce the need for adjunct imaging and microbiological assessments for accurate diagnosis. Overall, these additional studies complement our meta-analysis by reinforcing the significance of enterococcal infections, the role of surgical timing, and the diagnostic challenges associated with NVO + IE.

This study has several strengths, including the comprehensive synthesis of available data, the low risk of publication bias, and the rigorous methodological approach used for mortality estimation. However, limitations must be acknowledged.

Mortality at 3 years appeared numerically lower than at 1 year (16 % vs. 18 %), likely reflecting differences in the included studies at each time point rather than a true survival pattern. The apparent decrease in mortality between 1 and 3 years likely reflects differences in the studies contributing data at each time point and variable follow-up completeness, rather than a true decline in risk. This underscores the limitations of study-level aggregate data and supports the need for patient-level analyses to better capture long-term outcomes. Especially for NVO, ascertainment of cases varied across the studies since different definitions and diagnostic criteria were used (Petri et al., 2024; Petri et al., 2025). Heterogeneity was high across studies, reflecting differences in patient selection, treatment strategies, and follow-up duration. The lack of individual patient data prevented a more granular analysis of risk factors influencing long-term survival, such as MRSA infections, the role of early surgical intervention, or variations in antimicrobial regimens. Furthermore, some studies did not report all time points for mortality, limiting direct comparisons across cohorts. Conducting additional subgroup analyses was not feasible, as the included studies consistently presented data at the cohort level. This limitation may account for some of the observed heterogeneity and could affect part of the interpretability of the results. However, we were still able to perform a meta-regression, based on aggregated study-level data, not on individual patients. While imperfect, this allowed us to stratify studies in a reproducible manner. Another limitation is the classification of studies by predominant pathogen, which was based on even slight numerical differences between proportions of *S. aureus* vs. streptococci and may have led to misclassification in balanced cases. This pragmatic approach ensured consistent coding across studies with aggregate data. Finally, this analysis included only studies reporting 1-month mortality, chosen because it was the most reported endpoint across studies, and a clinical meaningful time point, potentially limiting generalizability.

In addition, most of the included studies were retrospective and exhibited substantial methodological limitations, including incomplete follow-up, lack of adjustment for confounding variables, and inconsistent outcome ascertainment. Although three prospective studies were included, their findings were consistent with retrospective cohorts and did not substantially alter the pooled estimates. Nonetheless, future prospective studies with standardized definitions and outcome reporting are essential to clarify the prognostic impact of concomitant NVO + IE. As reflected by the modified Newcastle–Ottawa scale, nearly half of the studies were judged to be of poor methodological quality, which contributed to downgrading the certainty of the evidence. The relatively small number of NVO + IE patients in several cohorts also increased statistical imprecision and magnified the influence of outlier studies. Another important limitation is the absence of a comparator cohort of patients with isolated NVO or IE. This precludes a direct assessment of whether the observed mortality is uniquely attributable to the coexistence of both conditions or simply reflects the additive burden of two severe infections. While some individual studies suggest a possible excess risk, pooled comparative data were not available, and future studies should explicitly address this question. Additionally, the temporal span of the included studies – some of which date back several decades – introduces variability in diagnostic standards, therapeutic approaches, and access to modern interventions. While the inclusion of older studies is an inherent feature of systematic reviews aimed at capturing all available evidence, it may affect the applicability of findings to contemporary practice. A subgroup analysis stratifying studies by publication date or era was considered but ultimately not feasible due to the small number of studies and limited sample size, which would have introduced further imprecision.

Collectively, these factors emphasize the need for standardized diagnostic criteria, prospective multi-center studies, and consistent outcome reporting to improve the reliability of future evidence in this complex clinical context.

Future research should focus on prospective studies to better define optimal diagnostic, antimicrobial, and surgical approaches in NVO + IE. A key area of investigation is whether early detection through advanced imaging (Maamari et al., 2023) and molecular diagnostics (Mahmoud et al., 2025) could reduce delays in treatment and improve survival. Additionally, further studies are needed to evaluate long-term functional outcomes, the role of combination antimicrobial therapy, and the potential benefits of standardized treatment protocols. Taken together, these observations highlight that the crucial unanswered question remains whether the concomitant occurrence of NVO and IE carries a worse prognosis than either infection alone. Only well-designed, prospective multi-center studies with harmonized diagnostic criteria can definitively address this gap.

## 5 Conclusions

In summary, this systematic review emphasizes the significant mortality impact linked to NVO + IE, especially for *S. aureus* etiology. The variability in reported outcomes points to the necessity for more clearly defined management approaches to enhance patient care. With the increasing prevalence of both conditions, prompt identification, customized antimicrobial treatment, and collaborative multidisciplinary intervention will be essential for improving survival rates and minimizing complications in this high-risk group.

## Supplement

10.5194/jbji-10-425-2025-supplement The supplement related to this article is available online at https://doi.org/10.5194/jbji-10-425-2025-supplement.

## Data Availability

The full dataset is available through the first and corresponding authors upon reasonable request.
