# Supplementary material for: Infective endocarditis meets native vertebral osteomyelitis: a mortality perspective
Source: J Bone Jt Infect. 2025 Nov 5;10(6):425–35. doi: 10.5194/jbji-10-425-2025 (PMC12606593; doi:10.5194/jbji-10-425-2025)
Supplement: The supplement related to this article is available online at https://doi.org/10.5194/jbji-10-425-2025-supplement. [file jbji-10-425-2025-supplement.pdf]

Supplement of J. Bone Joint Infect., 10, 425–435, 2025
https://doi.org/10.5194/jbji-10-425-2025-supplement
© Author(s) 2025. CC BY 4.0 License.

Supplement of

Infective endocarditis meets native vertebral osteomyelitis: a mortality perspective

Fabio Borgonovo et al.

Correspondence to: Francesco Petri (petri.francesco@mayo.edu) and Elie F. Berbari (berbari.elie@mayo.edu)

The copyright of individual parts of the supplement might differ from the article licence.

SUPPLEMENTARY MATERIALS

Content

- Funnel Plots for Publication Bias Assessment
- Methodological Quality Assessment
- Inclusion and Exclusion Criteria
- Data Extraction
- Search Strategy
- PRISMA 2020 Checklist
- Characteristics of the Included Studies
- Inter-rater Reliability
- Certainty of Evidence
- References

Figure S1 – Funnel plots showing Publication Bias across different timepoints for pooled mortality analyses

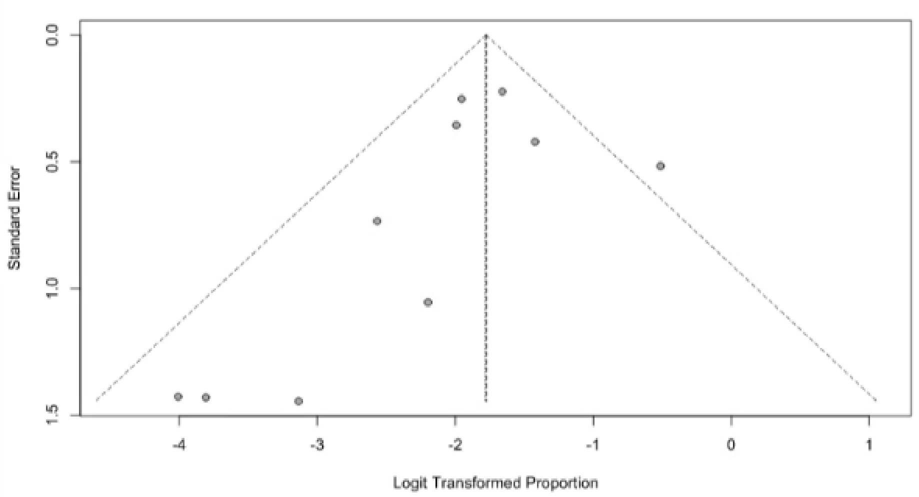

Funnel plot for in-hospital pooled mortality

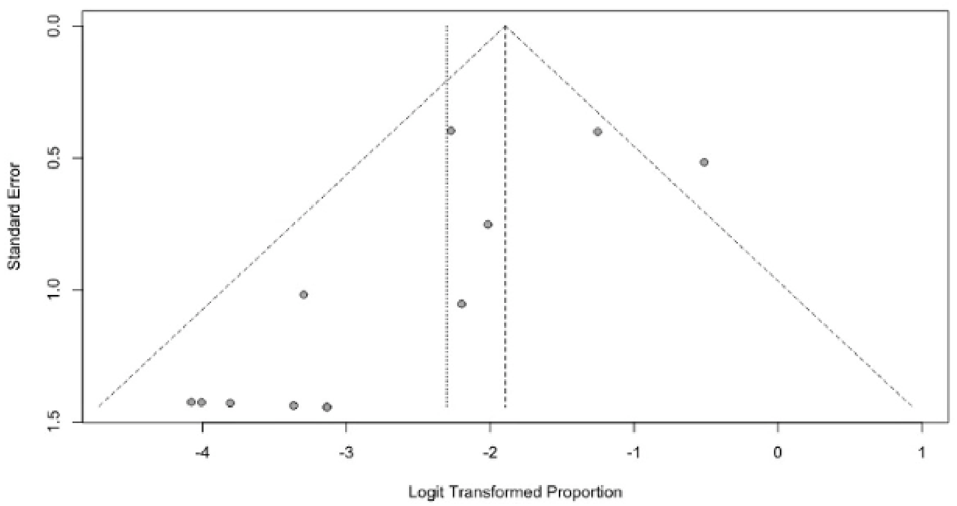

Funnel plot for 1-month pooled mortality

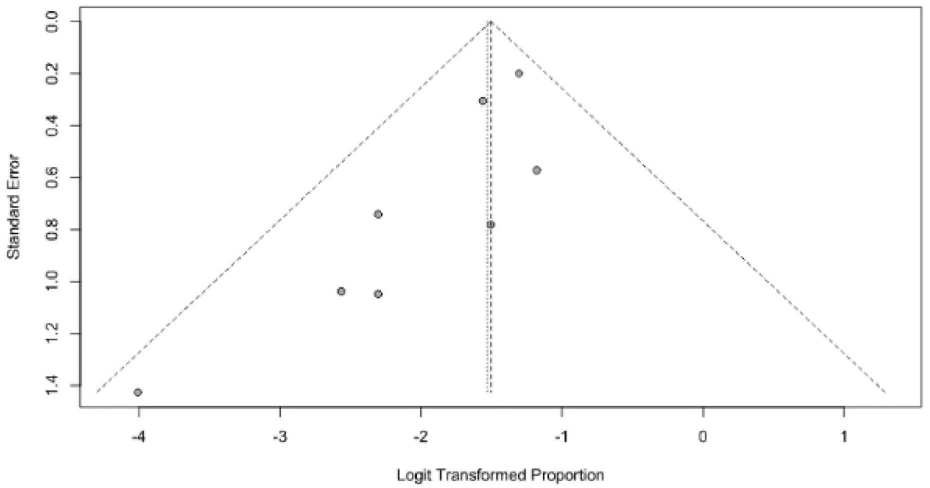

Funnel plot for 1-year pooled mortality

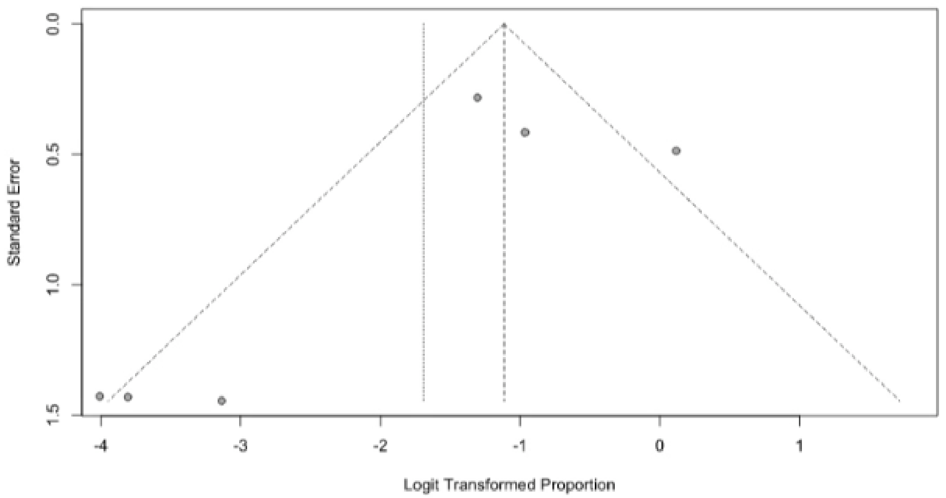

Funnel plot for 3-year pooled mortality

Table S1 – Methodological quality assessment for case series.

Author	Year	**Selection** – Does the patient(s) represent(s) the whole experience of the investigator (centre) or is the selection method unclear to the extent that other patients with similar presentation may not have been reported?	**Ascertainment** – Was the exposure adequately ascertained?	**Ascertainment** – Was the outcome adequately ascertained?	**Outcome** – Was follow-up long enough for outcomes to occur	**Reporting** – Is the case(s) described with sufficient details to allow other investigators to replicate the research or to allow practitioners make inferences related to their own practice?	**Overall**
Akiyama	2013	−	−	−	−	+	−
Behmanesh	2019	−	−	−	−	−	−
Carbone	2020	+	+	−	+	+	+
Del Pace	2021	−	−	+	+	+	+
Koslow	2014	+	+	+	+	−	+
Le Moal	2002	+	+	−	−	+	+
Mulleman	2006	−	−	−	−	−	−
Ninet	1984	−	−	−	−	−	−
Pigrau	2005	+	+	−	−	+	+

**Author**	**Year**	**Selection –** Does the patient(s) represent(s) the whole experience of the investigator (centre) or is the selection method unclear to the extent that other patients with similar presentation may not have been reported?	**Ascertainment –** Was the exposure adequately ascertained?	**Ascertainment –** Was the outcome adequately ascertained?	**Outcome –** Was follow-up long enough for outcomes to occur	**Reporting –** Is the case(s) described with sufficient details to allow other investigators to replicate the research or to allow practitioners make inferences related to their own practice?	
Tamura	2010	+	+	−	+	−	+
Viezens	2022	−	−	−	−	−	−
Castagne'	2021	+	+	+	+	−	+
Aguilar	2018	+	+	−	−	+	+
Pola	2018	+	+	+	+	+	+
Van Soest	2023	+	+	+	+	−	+
Saha	2023	+	−	−	−	−	−

Inclusion and Exclusion Criteria

Included:

- Randomized controlled trials (RCTs)
- Non-randomized studies: non-randomized controlled trials, consecutive case series and cohort studies
- No language restriction
- Adult patients - 18 years old and above
- Patients with infective endocarditis (IE) and natural vertebral osteomyelitis (NVO) coinfection n > 10

Excluded:

- Review articles, updates, consensus statements, letters, case reports, commentaries, narrative reviews, guidelines. These are not original studies
- Pediatric patients (age < 18 years old)
- Animal studies

Outcomes:

- Intra hospital dead
- Dead at 1 month
- Dead at 1 year
- Dead at 3 years

S2 - Data Extraction

From each included study, we extracted the following data: first author's last name, year of publication, country of origin, study design (prospective or retrospective), as well as the start and end years of patient enrollment. The original objective of the study, along with the inclusion and exclusion criteria, were also recorded.

Regarding the study population, we collected information on the total number of patients, the specific infection category under investigation—native vertebral osteomyelitis (NVO) or infective endocarditis (IE)—and the number of individuals with both conditions (NVO+IE). Baseline demographic variables included age (reported in years) and sex, expressed as the absolute number of female patients.

Mortality data were extracted at multiple timepoints, including deaths at 1 month, 1 year, and 3 years, in addition to in-hospital mortality. When reported, data were collected specifically for the subgroup of patients with concomitant NVO and IE.

Clinical characteristics for this subgroup were extracted in detail and included the presence of diabetes mellitus, immunosuppressive conditions, chronic renal failure, and predisposing heart conditions (excluding prosthetic valves and pacemakers). Additional comorbidities of interest included the presence of a prosthetic valve, pacemaker, or history of intravenous drug use

(PWID). Clinical complications and disease manifestations were also recorded, including whether the patient underwent surgery, developed embolic phenomena, or presented with an epidural abscess.

Microbiological and anatomical data included the causative pathogens (Staphylococcus aureus, Streptococcus spp., Enterococci, culture-negative cases, and other organisms) and the location of IE (aortic, mitral, or tricuspid valve involvement). Spinal involvement was detailed by vertebral level, categorizing cases as cervical, thoracic, or lumbar/sacral.

Lastly, the reported duration of treatment (in days) was extracted when available.

Search Strategy

Database(s): Ovid MEDLINE(R) 1946 to Present and Epub Ahead of Print, In-Process & Other Non-Indexed Citations and Ovid MEDLINE(R) Daily, EBM Reviews - Cochrane Central Register of Controlled Trials September 2023, EBM Reviews - Cochrane Database of Systematic Reviews 2005 to October 18, 2023, Embase 1974 to 2023 October 18

Table S2 - Search Strategy

#	Searches
1	Discitis/
2	Spondylitis/
3	(diskitis or discitis or discospondylitis or diskospondylitis or spondylodiscitis or spondylodiskitis or spondylitis or osteodiscitis).ti,ab,hw,kf.
4	Tuberculosis, Spinal/
5	or/1-4
6	Osteomyelitis/
7	exp *Bone Diseases, Infectious/ or exp *"bone and joint infections"/
8	*Infections/di
9	(infect* or septic or sepsis).ti.
10	osteomyelitis.ti,ab,hw,kf.
11	or/6-10
12	exp *Spine/
13	*Spinal Diseases/
14	(spinal or spine or spondy* or vertebra* or disc or disk or "disco-vertebral").ti.
15	or/12-14
16	11 and 15
17	((septic or sepsis or infection* or infectious or infective or tuberculosis) adj2 (spine or spinal or spondylitis or vertebr* or disc or discs or disk or disks or "disco-vertebral")).ti,ab.
18	5 or 16 or 17
19	exp endocarditis/
20	(endocardi* adj1 inflammat*).ti,ab,hw,kf.
21	endocarditis.ti,ab,hw,kf.
22	19 or 20 or 21
23	18 and 22
24	(exp animals/ or exp nonhuman/) not exp humans/
25	((alpaca or alpacas or algae* or amphibian or amphibians or animal or animals or antelope or armadillo or armadillos or avian or baboon or baboons or bats beagle or beagles or bee or bees or bird or birds or bison or bovine or buffalo or buffaloes or buffalos or "c elegans" or "Caenorhabditis elegans" or camel or camels or canine or canines or canis or carp or cats or catfish or cattle or chamaeleo* or chameleon* or chick or chicken or chickens or chicks or chimp or chimpanze or chimpanzees or chimps or cow or cows or "D melanogaster" or "dairy calf" or "dairy calves" or deer or dog or dogs or donkey or donkeys or drosophila or "Drosophila melanogaster" or duck or duckling or ducklings or ducks or equid or equids or equine or equines or feline or felines or ferret or ferrets or finch or finches or fish or flatworm or flatworms or fox or foxes or frog or frogs or "fruit flies" or "fruit fly" or "G mellonella" or "Galleria mellonella" or geese or gerbil or gerbils or goat or goats or goose or gorilla or gorillas or groundhog or groundhogs or hamster or hamsters or hare or hares [illegible]

	heifer or heifers or horse or horses or iguana or iguanas or insect or insects or jellyfish or kangaroo or kangaroos or kitten or kittens or "laboratory animal*" lagomorph or lagomorphs or lamb or lambs or lemur or lemurs or lemuridae or llama or llamas or macaque or macaques or macaw or macaws or marmoset marmosets or mice or minipig or minipigs or mink or minks or monkey or monkeys or mouse or mule or mules or muskrat or muskrats or nematode or nematodes or newt or newts or octopus or octopuses or orangutan or "orang-utan" or orangutans or "orang-utans" or oxen or parrot or parrots or pig or pigeo or pigeons or piglet or piglets or pigs or porcine or primate or primates or poultry or quail or rabbit or rabbits or rat or rats or reptile or reptiles or rodent or rodents or ruminant or ruminants or salmon or sheep or shrimp or slug or slugs or swine or tamarin or tamarins or tilapia or tilapias or toad or toads or trout urchin or urchins or vole or voles or waxworm or waxworms or weasel or weasels or wolf or wolves or worm or worms or wrass* or xenopus or "zebra fish zebrafish) not (human or humans or patient or patients)).ti,ab,hw,kw.
26	(rat or rats or mice or mouse or murine or pig or pigs or porcine or swine or dog or dogs).ti.
27	or/24-26
28	(conference abstract or conference review or editorial or erratum or note or addresses or autobiography or bibliography or biography or blogs or comment o dictionary or directory or interactive tutorial or interview or lectures or legal cases or legislation or news or newspaper article or patient education handout o periodical index or portraits or published erratum or video-audio media or webcasts).mp. or conference abstract.st.
29	23 not 27
30	29 not 28
31	remove duplicates from 30

SCOPUS

1	TITLE-ABS-KEY (diskitis OR discitis OR discospondylitis OR diskospondylitis OR spondylodiscitis OR spondylodiskitis OR spondylitis OR osteodisciti OR TITLE-ABS-KEY ((septic OR sepsis OR infection* OR infectious OR infective OR tuberculosis) W/2 (spine OR spinal OR spondylitis OR vertebr* OR disc OR discs OR disk OR disks OR "disco-vertebral"))
2	TITLE-ABS-KEY (endocardi* W/1 inflammat*) OR TITLE-ABS-KEY (endocarditis)
3	1 and 2
4	INDEX(embase) OR INDEX(medline) OR PMID(0* OR 1* OR 2* OR 3* OR 4* OR 5* OR 6* OR 7* OR 8* OR 9*)
5	3 not 4
6	(TITLE-ABS-KEY ((alpaca OR alpacas OR amphibian OR amphibians OR animal OR animals OR antelope OR armadillo OR armadillos OR avian OR baboon OR baboons OR beagle OR beagles OR bee OR bees OR bird OR birds OR bison OR bovine OR buffalo OR buffaloes O buffalos OR "c elegans" OR "Caenorhabditis elegans" OR camel OR camels OR canine OR canines OR carp OR cats OR cattle OR chick OR chicken OR chickens OR chicks OR chimp OR chimpanze OR chimpanzees OR chimps OR cow OR cows OR "D melanogaster" OR "dairy ca OR "dairy calves" OR deer OR dog OR dogs OR donkey OR donkeys OR drosophila OR "Drosophila melanogaster" OR duck OR duckling O ducklings OR ducks OR equid OR equids OR equine OR equines OR feline OR felines OR ferret OR ferrets OR finch OR finches OR fish flatworm OR flatworms OR fox OR foxes OR frog OR frogs OR "fruit flies" OR "fruit fly" OR "G mellonella" OR "Galleria mellonella" OR geese OR gerbil OR gerbils OR goat OR goats OR goose OR gorilla OR gorillas OR hamster OR hamsters OR hare OR hares OR heifer OR heifers OR horse OR horses OR insect OR insects OR jellyfish OR kangaroo OR kangaroos OR kitten OR kittens OR lagomorph OR lagomorphs OR lamb OR lambs OR llama OR llamas OR macaque OR macaques OR macaw OR macaws OR marmoset OR marmosets OR mice OR minipig OR minipigs OR mink OR minks OR monkey OR monkeys OR mouse OR mule OR mules OR nematode OR nematodes octopus OR octopuses OR orangutan OR "orang-utan" OR orangutans OR "orang-utans" OR oxen OR parrot OR parrots OR pig OR pigeon O pigeons OR piglet OR piglets OR pigs OR porcine OR primate OR primates OR quail OR rabbit OR rabbits OR rat OR rats OR reptile OR reptiles OR rodent OR rodents OR ruminant OR ruminants OR salmon OR sheep OR shrimp OR slug OR slugs OR swine OR tamarin OR tamarins OR toad OR toads OR trout OR urchin OR urchins OR vole OR voles OR waxworm OR waxworms OR worm OR worms OR xenopus OR "zebra fish" OR zebrafish) AND NOT (human OR humans OR patient OR patients)))
7	5 not 6
8	LIMIT-TO (DOCTYPE , "ar")

Table S3 – PRISMA 2020 Checklist

Section and Topic	Item #	Checklist item	Location where item is reported
**TITLE**			
Title	1	Identify the report as a systematic review.	Lines 1-2
**ABSTRACT**			
Abstract	2	See the PRISMA 2020 for Abstracts checklist.	Lines 24-44
**INTRODUCTION**			
Rationale	3	Describe the rationale for the review in the context of existing knowledge.	Lines 46-64
Objectives	4	Provide an explicit statement of the objective(s) or question(s) the review addresses.	Lines 65-66
**METHODS**			
Eligibility criteria	5	Specify the inclusion and exclusion criteria for the review and how studies were grouped for the syntheses.	Lines 75-81, Supplementary materials
Information sources	6	Specify all databases, registers, websites, organisations, reference lists and other sources searched or consulted to identify studies. Specify the date when each source was last searched or consulted.	Lines 83-89
Search strategy	7	Present the full search strategies for all databases, registers and websites, including any filters and limits used.	Supplementary materials
Selection process	8	Specify the methods used to decide whether a study met the inclusion criteria of the review, including how many reviewers screened each record and each report retrieved, whether they worked independently, and if applicable, details of automation tools used in the process.	Lines 91-100, Supplementary materials
Data collection process	9	Specify the methods used to collect data from reports, including how many reviewers collected data from each report, whether they worked independently, any processes for obtaining or confirming data from study investigators, and if applicable, details of automation tools used in the process.	Lines 102-110, Supplementary materials
Data items	10a	List and define all outcomes for which data were sought. Specify whether all results that were compatible with each outcome domain in each study were sought (e.g. for all measures, time points, analyses), and if not, the methods used to decide which results to collect.	Lines 116-120; Supplementary materials
	10b	List and define all other variables for which data were sought (e.g. participant and intervention characteristics, funding sources). Describe any assumptions made about any missing or unclear information.	Supplementary materials
Study risk of bias assessment	11	Specify the methods used to assess risk of bias in the included studies, including details of the tool(s) used, how many reviewers assessed each study and whether they worked independently, and if applicable, details of automation tools used in the process.	Lines 111-114; Supplementary materials
Effect measures	12	Specify for each outcome the effect measure(s) (e.g. risk ratio, mean difference) used in the synthesis or presentation of results.	Lines 116-120
Synthesis methods	13a	Describe the processes used to decide which studies were eligible for each synthesis (e.g. tabulating the study intervention characteristics and comparing against the planned groups for each synthesis (item #5)).	Lines 121-130; Supplementary materials
	13b	Describe any methods required to prepare the data for presentation or synthesis, such as handling of missing summary statistics, or data conversions.	Lines 121-130; Supplementary materials
	13c	Describe any methods used to tabulate or visually display results of individual studies and syntheses.	Lines 121-130; Supplementary materials
	13d	Describe any methods used to synthesize results and provide a rationale for the choice(s). If meta-analysis was performed, describe the model(s), method(s) to identify the presence and extent of statistical heterogeneity, and software package(s) used.	Lines 121-130; Supplementary materials
	13e	Describe any methods used to explore possible causes of heterogeneity among study results (e.g. subgroup analysis, meta-regression).	Lines 121-130; Supplementary materials
	13f	Describe any sensitivity analyses conducted to assess robustness of the synthesized results.	NA
Reporting bias assessment	14	Describe any methods used to assess risk of bias due to missing results in a synthesis (arising from reporting biases).	Lines 111-114; Supplementary materials
Certainty assessment	15	Describe any methods used to assess certainty (or confidence) in the body of evidence for an outcome.	Lines 111-114; Supplementary materials
**RESULTS**			
Study selection	16a	Describe the results of the search and selection process, from the number of records identified in the search to the number of studies included in the review, ideally using a flow	Lines 133-135; Figure 1

Section and Topic	Item #	Checklist item	Location where item is reported
		diagram.	
	16b	Cite studies that might appear to meet the inclusion criteria, but which were excluded, and explain why they were excluded.	Figure 1
Study characteristics	17	Cite each included study and present its characteristics.	Lines 135-142; Table 1; Supplementary materials
Risk of bias in studies	18	Present assessments of risk of bias for each included study.	Lines 186-188; Supplementary material and method
Results of individual studies	19	For all outcomes, present, for each study: (a) summary statistics for each group (where appropriate) and (b) an effect estimate and its precision (e.g. confidence/credible interval), ideally using structured tables or plots.	Lines 148-182; Tables 2-5
Results of syntheses	20a	For each synthesis, briefly summarise the characteristics and risk of bias among contributing studies.	Lines 186-188; Supplementary materials
	20b	Present results of all statistical syntheses conducted. If meta-analysis was done, present for each the summary estimate and its precision (e.g. confidence/credible interval) and measures of statistical heterogeneity. If comparing groups, describe the direction of the effect.	Tables 2-5
	20c	Present results of all investigations of possible causes of heterogeneity among study results.	Lines178-182; Table 6
	20d	Present results of all sensitivity analyses conducted to assess the robustness of the synthesized results.	NA
Reporting biases	21	Present assessments of risk of bias due to missing results (arising from reporting biases) for each synthesis assessed.	Supplementary materials
Certainty of evidence	22	Present assessments of certainty (or confidence) in the body of evidence for each outcome assessed.	Lines 186-188; Supplementary materials
**DISCUSSION**			
Discussion	23a	Provide a general interpretation of the results in the context of other evidence.	Lines 190-249
	23b	Discuss any limitations of the evidence included in the review.	Lines 250-282; page 12, lines 1-13
	23c	Discuss any limitations of the review processes used.	Lines 250-282
	23d	Discuss implications of the results for practice, policy, and future research.	Lines 283-289
**OTHER INFORMATION**			
Registration and protocol	24a	Provide registration information for the review, including register name and registration number, or state that the review was not registered.	NA
	24b	Indicate where the review protocol can be accessed, or state that a protocol was not prepared.	Lines 71-73
	24c	Describe and explain any amendments to information provided at registration or in the protocol.	NA
Support	25	Describe sources of financial or non-financial support for the review, and the role of the funders or sponsors in the review.	Title page
Competing interests	26	Declare any competing interests of review authors.	Title page
Availability of data, code and other materials	27	Report which of the following are publicly available and where they can be found: template data collection forms; data extracted from included studies; data used for all analyses; analytic code; any other materials used in the review.	Title page

Table S4 - Summary of baseline characteristics of all included studies.

First Author	Year	Design	Country	Study objective	Inclusion	Exclusion	Total n of patients	Patients with combined infection (NVO + IE)	Female n (%)	Age (mean / median)	In hospital deaths	Dead at 1 month	Dead at 1 year	Dead at 3 years
**Akiyama** et al. (1)	2013	R	Japan	To examine the incidence of vertebral osteomyelitis (VO) and the clinical features of VO	patients who were diagnosed with VO according to the following ICD-10-based codes: VO (M46.2), pyogenic infection of intervertebral disk (M46.3), unspecified discitis (M46.4), other infective spondylopathy (M46.5), other specified inflammatory spondylopathy (M46.8), unspecified inflammatory spondylopathy (M46.9), unspecified spondylopathy (M48.9), VT (A18.0 and M49.0), Brucella spondylitis (M49.1), enterobacterial spondylitis (M49.2) and spondylopathy in other infectious or parasitic diseases (M49.3).	NA	7118	145	NA	69.2	18	NA	NA	NA
**Behmanesh** et al. (2)	2019	P	Germany	To highlight the incidence of IE and the risk factors and clinical outcomes in patients with pyogenic spondylodiscitis	All patients admitted to the Department of Neurosurgery in Frankfurt with newly developed pyogenic spondylodiscitis were prospectively entered into an institutional database	NA	110	36	9	70.3	NA	8	NA	NA

**Carbone** et al. (3)	2020	P	France	to assess the incidence, epidemiology, clinical presentation, prognosis and therapeutic implications of PS in patients with IE	All patients hospitalized for suspected IE were accepted on admission to participate in this research protocol.	NA	1755	150	35	69	24	NA	32	NA
**Del Pace** et al. (4)	2021	R	Italy	evaluating the prevalence of definite SD in patients with IE	admitted to our department with a definite diagnosis of IE according to modified Duke University criteria, between January 2013 and December 2019.	NA	363	29	4	64.6	NA	0	NA	8
**Koslow** et al. (5)	2014	R	Israel	to characterize the rate, clinical features, and long-term outcome of infectious endocarditis among a cohort of patients with vertebral osteomyelitis	We reviewed all the patients with a clinical diagnosis of osteomyelitis identified from the computer database, a total of 690 patients. All patients with a clinical diagnosis of vertebral osteomyelitis proved by spinal imaging were selected for review, a total of 62 patients	NA	62	17	4	70.6	NA	2	4	9
**Le Moal** et al. (6)	2002	R	France	to evaluate the frequency of spondylodiscitis in patients with IE	Only patients with definite IE according to the duke criteria were included	NA	92	14	5	69.1	NA	NA	1	NA
**Mulleman** et al. (7)	2006	R	France	examined the clinical features of pyogenic spondylodiscitis due to streptococcal and enterococcal species	pyogenic spondylodiscitis (excluding postoperative cases) admitted from 1990 through 2002 to the Department of Rheumatology in Lille, a large metropolitan area of 1.1 million inhabitants,	NA	136	11	2	64.7	NA	0	2	NA

					were retrospectively reviewed and patients with SESD were identified.									
**Ninet** et al. (8)	1984	R	France	Our purpose, in the present study, was therefore characterize the clinical and bacteriological features of this association (NVO + IE)	Patients diagnosed NVO + IE. Von Reyn's criteria for IE. Diagnosis of NVO was made on clinical, roentgenographic and sometimes, bone scanning notions.	NA	430	14	2	56	NA	0	1	NA
**Pigrau** et al. (9)	2005	R	Spain	This study investigates the incidence and risk factors of infectious endocarditis in patients with pyogenic vertebral osteomyelitis, and the outcome of pyogenic vertebral osteomyelitis with and without associated infectious endocarditis.	We retrospectively reviewed all cases of vertebral osteomyelitis diagnosed at Vall d'Hebron Hospital, a 650-bed tertiary referral center, from January 1986 to June 2002. During the study period, all patients with bacteremia, and particularly those with endocarditis, were evaluated by infectious disease staff members, and 86 of 91 patients with pyogenic vertebral osteomyelitis were seen and followed up by one of the authors.	Patients with prior spinal instrumentation or surgery and those with tuberculosis (n 19), brucellosis (n 9), or no definitive bacteriologic diagnosis (n 11) were excluded.	91	28	7	66	2	1	NA	NA
**Tamura** et al. (10)	2010	R	Japan	The purpose of this study was to investigate the incidence, the clinical features, and the outcome for VO in patients with IE.	Only patients with definite IE according to the Durack criteria were included	NA	58	11	3	61.2	0	0	1	0

**Viezens** et al. (11)	2022	P	Germany	We therefore aimed to use a prospectively managed data-base to analyze the influence of a simultaneously present IE in patients with already diagnosed spondylodiscitis in regard to differences in clinical care. We furthermore aimed to develop a novel treatment algorithm to be able to standardize diagnostic as well as therapeutic strategies in this cohort of patients.	all patients treated with proven spontaneous spondylodiscitis at a tertiary centre were included in a prospective database.	NA	328	36	11	65.8	7	NA	NA	NA
**Castagne'** et al. (12)	2021	R	France	The main objective of the study was to compare the relapse rate at 1 year in patients with infective endocarditis-associated with vertebral osteomyelitis and patients with vertebral osteomyelitis alone.	The inclusion criteria were patients hospitalized at the Clermont-Ferrand University Hospital for infectious vertebral osteomyelitis, defined by bacteriological documentation (blood culture and/or disco-vertebral biopsy) and compatible imaging (MRI or CT scan), between 01/01/2014 and 31/12/2017.	Non-inclusion criteria were no data available at 1 year (lost to follow-up, death not related to recurrence/relapse), verte-bral osteomyelitis without microbiological documentation/post-operative/spine with material, vertebral osteomyelitis treated more than 6	62	27	7	71.9	0	0	0	0

						weeks, pacemaker infections, and patients requiring vasopressor amines.							
**Aguilar** et al.(13)	2018	R	Spain	To describe the demographic, clinical, and microbiological profile of native vertebral osteomyelitis (NVO) in aged patients as compared to that of younger patients, to identify differences that could motivate changes in clinical management	All adult patients (18 years or older) with a microbiologically confirmed diagnosis of NVO treated at our center from 1990 to 2015 were enrolled.	NA	247	75	23	69	9	7	13	16
**Pola** et al. (14)	2018	R	Italy	Aim of this study was to describe the clinical features of PS and to evaluate the prognostic factors and the long-term outcomes of a large population of patients.	All consecutive cases of PS treated in a 1100-bed univer-sity hospital over a 9-year period (2008–2016) with a 2-year follow-up were enrolled.	Suspected or confirmed non-pyogenic spondylodiscitis (e.g. tubercular and brucellar infections) were excluded.	207	22	2	64	0	0	2	0
**Van Soest** et al. (15)	2023	Mixed*	Denmark	Staphylococcus aureus is an uncommon cause of community-acquired bacterial meningitis. We aimed to describe patients with this disease.	This study includes patients with community-acquired bacterial meningitis, aged 16 years or older, to investigate host genetic risk factors for bacterial menin- gitis. Procedures of inclusion were discussed in detail previously.	Patients were excluded if they were categorized as hospital-acquired meningitis defined as meningitis developing > 48 h	111	10	5	66	1	1	NA	NA

						after admission, or within a week af- ter discharge. All patients who underwent a neurosurgical proce- dure or a significant head trauma within one month of the menin- gitis episode, as well as those with a neurosurgical device in situ were excluded.							
**Saha** et al**.** (16)	2023	R	Germany	We reviewed all patients who underwent cardiac surgery for IE at our institution with a focus on causative organisms and infective foci.	patients underwent cardiac surgery at our center; this in-cluded 160 patients (4%) who were operated due to IE.	Patients with pacemaker infection without indication for heart valve surgery were excluded from the study.	160	16	3	74	6	6	NA	NA

Abbreviations: **R**: retrospective cohort study; **P**: prospective cohort study; **NA**: not available.

* "For both cohorts, data was collected prospectively through an online case record form […]. Missing data and data on co-infections, clinical indicators for endocarditis and spondylodiscitis, antibiotic usage and surgical treatment were completed retrospectively through review of clinical charts or discharge letters."

Inter-rater reliability (single average observed agreement percentage): 41.3%

Table S5: Certainty of Evidence

Certainty assessment							№ of patients		Effect		Certainty	Importance
№ of studies	Study design	Risk of bias	Inconsistency	Indirectness	Imprecision	Other considerations	standard medical therapy (antibiotics ± surgery)	no comparator	Relative (95% CI)	Absolute (95% CI)		
**In-hospital mortality (assessed with: Observed deaths among hospitalized patients)**												
10	non-randomised studies	serious[a]	not serious	not serious	seriousb	none	67/520 (12.9%)	none	not estimable	14.0% (CI: 10.0-20.0)	LOW [a,b]	CRITICAL
**1-month mortality (follow-up: 30 days; assessed with: Observed deaths within 30 days)**												
12	non-randomised studies	serious[c]	not serious	not serious	serious[d]	none	25/296 (8.4%)	none	not estimable	9.0% (CI:5.0–17.0)	LOW [c,d]	CRITICAL
**1-year mortality (follow-up: 12 months; assessed with: Observed deaths at 12 months)**												
9	non-randomised studies	serious[e]	not serious	not serious	serious[f]	none	56/341 (16.4%)	none	not estimable	18.0% (CI: 13.0–24.0)	LOW [e,f]	CRITICAL
**3-year mortality (follow-up: 36 months; assessed with: Observed deaths at 36 months)**												
6	non-randomised studies	serious[g]	serious[h]	not serious	serious[i]	none	33/181 (18.2%)	none	not estimable	16.0% (CI: 3.0–50.0)	VERY LOW [g,h,i]	CRITICAL

CI: confidence interval

Explanations

a. More than half of the studies had low quality methodology based on modified NOS (see Table S1)

b. 67 deaths only; wide CI; upper/lower bounds suggest uncertainty.

c. Most studies retrospective; Methodological assessment rated of low quality.

d. Low number of events and wide CI.

e. Majority retrospective, with unclear follow-up or confounding adjustment

f. Low number of events (56), wide CI.

g. Retrospective design, limited control of confounding, missing data risk

h. High variability in rates and high heterogeneity (70%).

i. Very few total events (33); CI wide (4.3–24.8%)

References:

1. Akiyama T, Chikuda H, Yasunaga H, Horiguchi H, Fushimi K, Saita K. Incidence and risk factors for mortality of vertebral osteomyelitis: a retrospective analysis using the Japanese diagnosis procedure combination database. BMJ Open. 2013 Mar 25;3(3):e002412.

2. Behmanesh B, Gessler F, Schnoes K, Dubinski D, Won SY, Konczalla J, et al. Infective endocarditis in patients with pyogenic spondylodiscitis: implications for diagnosis and therapy. Neurosurg Focus. 2019 Jan 1;46(1):E2.

3. Carbone A, Lieu A, Mouhat B, Santelli F, Philip M, Bohbot Y, et al. Spondylodiscitis complicating infective endocarditis. Heart Br Card Soc. 2020 Dec;106(24):1914–8.

4. Del Pace S, Scheggi V, Virgili G, Caciolli S, Olivotto I, Zoppetti N, et al. Endocarditis with spondylodiscitis: clinical characteristics and prognosis. BMC Cardiovasc Disord. 2021 Apr 15;21(1):186.

5. Koslow M, Kuperstein R, Eshed I, Perelman M, Maor E, Sidi Y. The unique clinical features and outcome of infectious endocarditis and vertebral osteomyelitis co-infection. Am J Med. 2014 Jul;127(7):669.e9-669.e15.

6. Le Moal G, Roblot F, Paccalin M, Sosner P, Burucoa C, Roblot P, et al. Clinical and laboratory characteristics of infective endocarditis when associated with spondylodiscitis. Eur J Clin Microbiol Infect Dis Off Publ Eur Soc Clin Microbiol. 2002 Sep;21(9):671–5.

7. Mulleman D, Philippe P, Senneville E, Costes C, Fages L, Deprez X, et al. Streptococcal and enterococcal spondylodiscitis (vertebral osteomyelitis). High incidence of infective endocarditis in 50 cases. J Rheumatol. 2006 Jan;33(1):91–7.

8. Ninet J, Gayet JL, Etienne J, Bonvoisin B, Vignon E, Berthou JD, et al. Bacterial endocarditis presenting as acute vertebral osteomyelitis: 14 cases. Eur Heart J. 1984 Oct 1;5(suppl_C):101–5.

9. Pigrau C, Almirante B, Flores X, Falco V, Rodríguez D, Gasser I, et al. Spontaneous pyogenic vertebral osteomyelitis and endocarditis: incidence, risk factors, and outcome. Am J Med. 2005 Nov;118(11):1287.

10. Tamura K. Clinical characteristics of infective endocarditis with vertebral osteomyelitis. J Infect Chemother Off J Jpn Soc Chemother. 2010 Aug;16(4):260–5.

11. Viezens L, Dreimann M, Strahl A, Heuer A, Koepke LG, Bay B, et al. Spontaneous spondylodiscitis and endocarditis: interdisciplinary experience from a tertiary institutional case series and proposal of a treatment algorithm. Neurosurg Rev. 2022 Apr;45(2):1335–42.

12. Castagné B, Soubrier M, Prouteau J, Mrozek N, Lesens O, Tournadre A, et al. A six-week antibiotic treatment of endocarditis with spondylodiscitis is not associated with increased risk of relapse: A retrospective cohort study. Infect Dis Now. 2021 May;51(3):253–9.

13. Aguilar-Company J, Pigrau C, Fernández-Hidalgo N, Rodríguez-Pardo D, Falcó V, Lung M, et al. Native vertebral osteomyelitis in aged patients: distinctive features. An observational cohort study. Infection. 2018 Oct 1;46(5):679–86.

14. Pola E, Taccari F, Autore G, Giovannenze F, Pambianco V, Cauda R, et al. Multidisciplinary management of pyogenic spondylodiscitis: epidemiological and clinical features, prognostic factors and long-term outcomes in 207 patients. Eur Spine J. 2018 Jun 1;27(2):229–36.

15. van Soest TM, Søndermølle MB, Brouwer MC, Chekrouni N, Larsen AR, Petersen A, et al. Community-acquired *Staphylococcus aureus* meningitis in adults. J Infect. 2023 Mar 1;86(3):239–44.

16. Saha S, Dudakova A, Danner BC, Kutschka I, Schulze MH, Niehaus H. Bacterial Spectrum and Infective Foci in Patients Operated for Infective Endocarditis: Time to Rethink Strategies? Thorac Cardiovasc Surg. 2022 Feb 8;71:02–11.
